# Systemic alterations of tricarboxylic acid cycle enzymes in Alzheimer’s disease

**DOI:** 10.3389/fnins.2023.1206688

**Published:** 2023-07-27

**Authors:** Dongdong Jia, Fangzhou Wang, Haitao Yu

**Affiliations:** ^1^The Affiliated Mental Health Center of Jiangnan University, Wuxi Central Rehabilitation Hospital, Wuxi, Jiangsu, China; ^2^Department of Fundamental Medicine, Wuxi School of Medicine, Jiangnan University, Wuxi, Jiangsu, China

**Keywords:** Alzheimer’s disease, tricarboxylic acid (TCA) cycle, transcriptomics, brain, peripheral blood cells

## Abstract

Mitochondrial dysfunction, especially tricarboxylic acid (TCA) cycle arrest, is strongly associated with Alzheimer’s disease (AD), however, its systemic alterations in the central and peripheral of AD patients are not well defined. Here, we performed an integrated analysis of AD brain and peripheral blood cells transcriptomics to reveal the expression levels of nine TCA cycle enzymes involving 35 genes. The results showed that TCA cycle related genes were consistently down-regulated in the AD brain, whereas 11 genes were increased and 16 genes were decreased in the peripheral system. Pearson analysis of the TCA cycle genes with Aβ, Tau and mini-mental state examination (MMSE) revealed several significant correlated genes, including pyruvate dehydrogenase complex subunit (PDHB), isocitrate dehydrogenase subunits (IDH3B, IDH3G), 2-oxoglutarate dehydrogenase complex subunit (DLD), succinyl-CoA synthetase subunit (SUCLA2), malate dehydrogenase subunit (MDH1). In addition, SUCLA2, MDH1, and PDHB were also uniformly down-regulated in peripheral blood cells, suggesting that they may be candidate biomarkers for the early diagnosis of AD. Taken together, TCA cycle enzymes were systemically altered in AD progression, PDHB, SUCLA2, and MDH1 may be potential diagnostic and therapeutic targets.

## Introduction

Alzheimer’s disease (AD) is the most common neurodegenerative disease, and its main clinical manifestations are cognitive impairment and progressive decline of memory ability (GBD 2016 Neurology Collaborators, 2019). The main pathology of AD includes amyloid (Aβ) plaque deposition and neurofibrillary tangles (NFTs) formed by hyperphosphorylation of Tau (Hodson, 2018; Scheltens et al., 2021). With the acceleration of population aging, the prevalence of AD has increased dramatically. In 2018, Alzheimer’s Disease International (ADI) estimated that there were about 50 million people with dementia worldwide, and it is expected that the global prevalence will increase by 2 times by 2050 (Scheltens et al., 2021), but the clinical treatment of AD is rarely effective. Therefore, there is an urgent need to find new regulatory molecules and the mechanisms for AD. There has been clear evidence that brain glucose metabolism is significantly reduced before cognitive decline (Cunnane et al., 2011; Ding et al., 2013), with positron emission tomography (PET) imaging showing a 20–30% reduction in brain glucose utilization of AD patients (de Leon et al., 1983).

Neurons have a high energy demand and are mainly operated by mitochondria, whose defects lead to synaptic dysfunction, neuroinflammation and neuronal death (Shoshan-Barmatz et al., 2018). Electron transport chain (ETC) and tricarboxylic acid (TCA) cycle are the most important metabolic pathways in mitochondria. The TCA cycle can completely oxidize acetyl-CoA and provide electron carriers for the oxidative phosphorylation pathway (Butterfield and Halliwell, 2019). Therefore, the dysregulation of TCA cycle metabolic pathways may be closely related to the insufficient energy supply in AD brain. Previous studies on the expression levels of TCA cycle enzymes were mostly limited to brain tissue samples, and the sample size was small, making the validity of the data insufficient (Bubber et al., 2005; Sang et al., 2022).

In order to systematically elucidate the relationship between TCA cycle and energy metabolism in AD and find the key molecules involved, we integrated the transcriptome data of brain tissue (Xu et al., 2018) (Alzdata; AD = 269, Ctrl = 271)^1^ and peripheral blood cells (Sood et al., 2015) (GSE63060: Ctrl = 104, MCI = 80, AD = 145; GSE63061: Ctrl = 134, MCI = 109, AD = 139), and systematically analyzed the changes of TCA cycle enzymes in the central and peripheral tissues. By Pearson analysis, we identified genes correlated with Aβ and Tau pathology, as well as mini-mental state examination (MMSE), while PDHB, SUCLA2, and MDH1 may be potential diagnostic and therapeutic targets.

## Materials and methods

### Data acquisition

All datasets used to explore the expression of TCA cycle enzymes in the brain and periphery of AD patients are from the National Biotechnology Information Center (NCBI) Gene Expression Comprehensive Database (GEO) (Barrett et al., 2013). Specifically, brain data were obtained from 20 GSE series dataset, which have been integrated in the Alzdata database (see text footnote 1) (Xu et al., 2018), for a total of 269 AD patients and 271 normal aging subjects, including four brain regions [entorhinal cortex (EC): 39 vs. 39, hippocampus (HP): 74 vs. 65, temporal cortex (TC): 52 vs. 39, and frontal cortex (FC): 104 vs. 128]. Alzdata database has integrated and normalized multiple datasets across platforms, providing a reliable data resource for the exploration of pathological mechanisms and drug development related to AD. Specifically, individual expression datasets for each brain region were normalized, log2 transformed, and then merged by the compact algorithm in the ComBat R package to eliminate batch effects (Xu et al., 2018). Peripheral blood cells transcriptomes were obtained from GSE63060 (Ctrl = 104, MCI = 80, AD = 145) and GSE63061 (Ctrl = 134, MCI = 109, AD = 139), which were two large European blood cells RNA expression datasets, and contributing to find reliable peripheral biomarkers for AD diagnosis. Differentially expressed genes (DEGs) were identified using limma package in R, which included linear regression analysis adjusted for age and gender. The mRNA expression profile and Aβ/Tau pathological scores in AD model mice were obtained from Mouse Dementia Network (Matarin et al., 2015).^2^ The data required for MMSE correlation analysis were obtained from GSE1297.

### Correlation of potential genes with Aβ, Tau and MMSE

To confirm the association between brain biomarkers and Aβ/Tau pathology, we performed correlation analysis between the mRNA expression of related genes and Aβ/Tau pathology scores in AD mouse brain. Specifically, immunofluorescence was applied to perform Aβ_40_ and phosphorylated Tau semi-quantitative analysis, of which 56 brain tissue samples with Aβ pathological grading, and 14 brain tissue samples with Tau pathological grading. In addition, to confirm the association between brain biomarkers and cognitive decline, we performed correlation analysis between mRNA expression of related genes and MMSE scores in AD patients (GSE1297: Ctrl = 9, MCI = 22). Graphpad prism software (Graphpad software, La Jolla, CA, USA) was used for Pearson correlation analysis between gene mRNA expression and Aβ/Tau pathology, MMSE scores.

### Statistical analysis

Differentially expressed genes (DEGs) were identified using limma package in R,^3^ which included linear regression analysis adjusted for age and gender, and *P*-values < 0.05 was considered to be significant.

## Results

### The mRNA expression levels of TCA cycle genes in brain tissues from 269 AD patients and 271 controls

**FIGURE 1:**
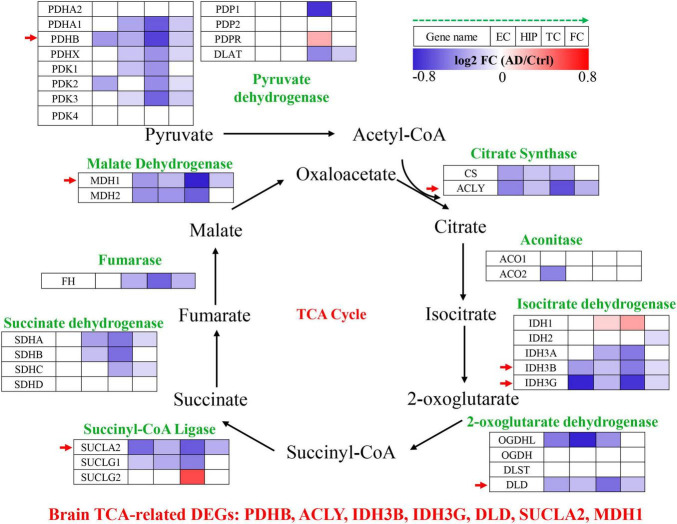
Tricarboxylic acid (TCA) cycle related genes were significantly differentially expressed in AD brain tissues. The increased (red) or decreased (blue) level of proteins in AD vs. Ctrl (as indicated by the green arrow, EC, HIP, TC, and FC from left to right, *P* < 0.05). EC, entorhinal cortex; HIP, hippocampus; TC, temporal cortex; FC, frontal cortex. Red arrows indicate genes that were consistently differentially expressed in the four brain regions.

The TCA cycle consists 35 genes, forming 9 key enzyme complexes that collectively drive the conversion of pyruvate to acetyl-CoA and complete oxidation. Brain data were obtained from the Alzdata database (see text footnote 1) (Xu et al., 2018), for a total of 269 AD patients and 271 normal aging subjects, including four brain regions (entorhinal cortex, hippocampus, temporal cortex, and frontal cortex). The mRNA expression levels of TCA cycle genes in brain tissues were shown in Figure 1 and Table 1.

**TABLE 1:** The mRNA expression levels of TCA cycle genes in brain tissues from 269 AD patients and 271 controls.

Brain region	Entorhinal cortex			Hippocampus			Temporal cortex			Frontal cortex		
Gene	log2 FC	*p*-value	adj *p*-value	log2 FC	*p*-value	adj *p*-value	log2 FC	*p*-value	adj *p*-value	log2 FC	*p*-value	adj *p*-value
**Pyruvate dehydrogenase**												
PDHA2	0.17	1.81E-01	3.57E-01	0.22	5.80E-02	2.20E-01	0.09	7.28E-01	8.59E-01	0.07	4.72E-01	8.34E-01
PDHA1	-0.11	1.95E-01	3.74E-01	-0.32	3.24E-05	3.00E-03	-0.59	5.96E-05	1.00E-03	-0.2	4.32E-04	6.00E-03
PDHB	-0.37	4.00E-03	3.70E-02	-0.3	1.00E-03	1.60E-02	-0.68	2.97E-05	1.00E-03	-0.2	1.00E-03	1.00E-02
PDHX	-0.15	1.37E-01	2.99E-01	-0.21	1.70E-02	1.07E-01	-0.39	1.00E-03	1.00E-02	-0.15	3.20E-02	1.05E-01
PDK1	-0.14	2.15E-01	3.97E-01	-0.22	5.00E-03	5.50E-02	-0.39	9.00E-03	4.50E-02	-0.04	5.69E-01	7.14E-01
PDK2	-0.32	5.00E-03	3.80E-02	-0.09	3.64E-01	6.14E-01	-0.41	1.59E-04	3.00E-03	-0.12	2.90E-02	9.90E-02
PDK3	-0.14	3.15E-01	5.04E-01	-0.14	8.00E-02	2.64E-01	-0.56	2.74E-05	1.00E-03	-0.19	2.20E-02	3.38E-01
PDK4	0.22	3.04E-01	4.94E-01	0.11	4.68E-01	6.97E-01	0.35	1.25E-01	2.97E-01	0.09	4.69E-01	6.29E-01
PDP1	NA	NA	NA	-0.13	1.76E-01	4.13E-01	-0.74	1.27E-04	2.00E-03	-0.05	7.23E-01	9.27E-01
PDP2	-0.22	2.43E-01	4.27E-01	0.07	6.30E-01	8.10E-01	0.04	8.68E-01	9.38E-01	NA	NA	NA
Pyruvate dehydrogenase phosphatase regulatory subunit (PDPR)	0.12	2.71E-01	4.59E-01	-0.07	5.27E-01	7.40E-01	0.26	2.30E-02	9.00E-02	0.06	4.05E-01	5.70E-01
DLAT	-0.14	3.94E-01	5.80E-01	-0.14	1.12E-01	3.22E-01	-0.43	5.72E-05	1.00E-03	-0.19	9.00E-03	4.60E-02
**Citrate synthase**												
CS	-0.34	1.00E-03	1.20E-02	-0.21	1.00E-03	1.40E-02	-0.27	1.50E-02	6.80E-02	-0.03	5.37E-01	6.87E-01
ACLY	-0.44	3.00E-03	2.90E-02	-0.23	2.00E-03	3.10E-02	-0.63	4.90E-05	1.00E-03	-0.28	1.83E-05	1.00E-03
**Aconitase**												
ACO1	0.01	9.11E-01	9.49E-01	-0.12	2.21E-01	4.69E-01	0.15	2.18E-01	4.28E-01	-0.03	3.74E-01	5.37E-01
ACO2	-0.45	4.50E-05	3.00E-03	-0.17	1.39E-01	3.63E-01	-0.24	1.45E-01	3.29E-01	-0.08	1.49E-01	2.92E-01
**Isocitrate dehydrogenase**												
IDH1	0	9.64E-01	9.81E-01	0.14	3.20E-02	1.58E-01	0.29	1.30E-02	6.00E-02	0.09	6.20E-02	1.65E-01
IDH2	-0.13	2.83E-01	4.71E-01	-0.12	3.07E-01	5.60E-01	0.06	5.96E-01	7.75E-01	-0.13	4.70E-02	1.37E-01
IDH3A	-0.19	5.20E-02	1.62E-01	-0.3	1.00E-03	1.80E-02	-0.48	1.71E-04	3.00E-03	-0.08	1.37E-01	2.77E-01
IDH3B	-0.36	2.00E-03	2.10E-02	-0.23	2.00E-03	2.60E-02	-0.47	3.72E-05	1.00E-03	-0.13	2.60E-02	9.20E-02
IDH3G	-0.79	7.72E-06	1.00E-03	-0.26	6.00E-03	5.70E-02	-0.73	1.65E-07	4.32E-05	-0.15	2.00E-03	2.00E-02
**2-oxoglutarate dehydrogenase**												
OGDHL	-0.48	4.69E-04	1.00E-02	-0.79	8.03E-07	1.00E-03	-0.4	1.50E-02	6.50E-02	-0.14	9.30E-02	2.11E-01
OGDH	0.19	1.20E-01	2.74E-01	-0.15	1.36E-01	3.59E-01	0	9.87E-01	9.95E-01	0.08	2.03E-01	3.60E-01
DLST	NA	NA	NA	-0.11	8.80E-02	2.79E-01	0.01	8.86E-01	9.46E-01	-0.04	5.24E-01	8.59E-01
DLD	-0.32	2.60E-02	1.07E-01	-0.24	5.00E-03	5.50E-02	-0.52	6.80E-05	1.00E-03	-0.23	2.00E-03	2.00E-02
**Succinyl-CoA ligase**												
SUCLA2	-0.54	3.00E-03	2.70E-02	-0.28	9.00E-03	7.50E-02	-0.59	1.00E-03	8.00E-03	-0.29	3.00E-03	2.30E-02
SUCLG1	-0.21	3.60E-02	1.31E-01	-0.3	1.97E-05	2.00E-03	-0.46	1.00E-03	6.00E-03	-0.05	5.44E-01	6.93E-01
SUCLG2	NA	NA	NA	0.07	4.52E-01	6.86E-01	0.57	1.00E-03	1.20E-02	0.1	3.82E-01	7.87E-01
**Succinate dehydrogenase**												
SDHA	-0.14	1.21E-01	2.77E-01	-0.34	1.00E-03	1.70E-02	-0.49	1.10E-04	2.00E-03	-0.15	2.00E-03	1.90E-02
SDHB	-0.16	8.90E-02	2.26E-01	-0.23	2.40E-02	1.32E-01	-0.52	2.00E-03	1.30E-02	-0.06	3.35E-01	5.00E-01
SDHC	-0.02	7.96E-01	8.81E-01	-0.08	2.44E-01	4.94E-01	-0.31	4.00E-03	2.60E-02	-0.14	2.00E-03	1.50E-02
Succinate dehydrogenase [ubiquinone] cytochrome b small subunit (SDHD)	-0.05	6.48E-01	7.84E-01	-0.03	7.26E-01	8.66E-01	-0.15	2.80E-01	5.02E-01	0.02	7.53E-01	8.46E-01
**Fumarase**												
FH	-0.08	4.48E-01	6.28E-01	-0.29	4.00E-03	4.40E-02	-0.56	6.61E-05	1.00E-03	-0.27	4.79E-04	1.22E-01
**Malate dehydrogenase**												
MDH1	-0.37	5.00E-03	3.80E-02	-0.25	4.00E-03	4.30E-02	-0.8	3.94E-06	2.40E-04	-0.21	2.20E-02	8.30E-02
MDH2	-0.4	1.00E-03	1.80E-02	-0.38	7.97E-06	1.00E-03	-0.57	1.79E-05	1.00E-03	-0.14	5.90E-02	1.60E-01

The mRNA expression levels of TCA cycle genes in brain tissues were obtained from the Alzdata database (http://www.alzdata.org/) (Xu et al., 2018). Log2FC, log2 fold change.

Pyruvate dehydrogenase complex (PDHC) mainly promotes the conversion of pyruvate to acetyl-CoA, which then enters the tricarboxylic acid cycle (Seifert et al., 2007). Pyruvate dehydrogenase E1 component subunit α and β (PDHA1, PDHB), pyruvate dehydrogenase protein X component (PDHX), [pyruvate dehydrogenase (acetyl-transferring)] kinase isozyme (PDK1, PDK2, PDK3), and dihydrolipoyllysine S-Acetyltransferase (DLAT) were significantly decreased in multiple brain regions of AD. PDHB showed the greatest perturbation, which was significantly decreased in EC, HIP, TC and FC. Citrate synthase [CS, ATP-citrate synthase (ACLY)] acts synergistically to regulate the conversion balance of citrate to oxaloacetate and acetyl-CoA (Marin-Garcia et al., 1998; Potapova et al., 2000), which were also significantly reduced in multiple brain regions of AD. Aconitase (ACO1, ACO2) promoted citrate to isocitrate conversion, which were not changed significantly in AD brain, only ACO2 was decreased significantly in EC region. Isocitrate dehydrogenase mainly including IDH1, IDH2, IDH3A, IDH3B, IDH3G were significantly altered in AD brain. IDH1 was significantly elevated in HIP and TC brain regions, and IDH2 was significantly decreased only in FC brain region. Notably, both IDH3B and IDH3G were significantly decreased in the four brain regions EC, HIP, TC and FC. Moreover, 2-oxoglutarate dehydrogenase (OGDHL, DLD), succinyl-CoA ligase (SUCLA2, SUCLG1), succinate dehydrogenase [succinate dehydrogenase [ubiquinone] flavoprotein subunit (SDHA), succinate dehydrogenase [ubiquinone] iron-sulfur subunit (SDHB), SDHC], fumarase (FH), and malate dehydrogenase (MDH1, MDH2) were significantly decreased in multiple brain regions of AD.

Altogether, TCA cycle related genes were consistently down-regulated in the AD brain, PDHB, ACLY, IDH3B, IDH3G, DLD, SUCLA2, and MDH1 were significantly decreased in the four brain regions EC, HIP, TC and FC, which may be potential brain biomarkers (Figure 1).

### Correlation of brain biomarkers with Aβ and Tau pathology in AD mouse models

**FIGURE 2:**
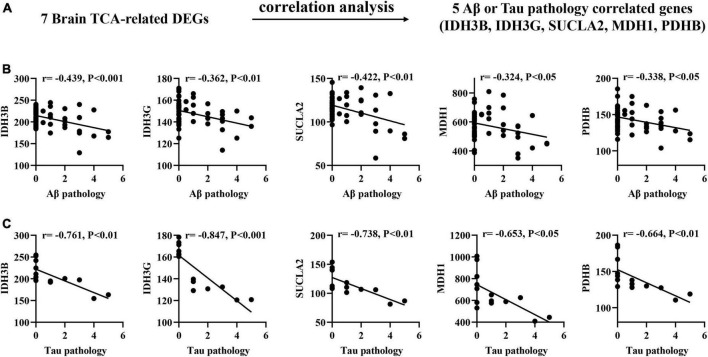
Correlation of brain TCA-related DEGs with Aβ and Tau pathology in AD mouse models. **(A)** Correlation analysis gene determination process **(B,C)** Pearson correlation coefficients (*r*) and corresponding *P*-values < 0.05 or 0.01 were displayed at the top of each plot. *X*-axis shows Aβ or Tau pathology score, *y*-axis indicates relative expression abundance of each gene.

To determine the association between brain biomarkers and AD progression, we analyzed the expression of brain biomarkers in AD mice and correlated them with Aβ and Tau pathology (Figure 2A). Pearson correlation analysis found that the IDH3B, IDH3G, SUCLA2, MDH1, PDHB showed moderate negative correlations with Aβ pathology (| *r*| = 0.189–0.439, *p* < 0.05; Figure 2B), and strong negative correlations with Tau pathology (| *r*| = 0.653–0.847, *p* < 0.05; Figure 2C), implying that their reduction significantly exacerbates AD pathology.

### Correlation of brain TCA-related DEGs with MMSE scores in AD patients

**FIGURE 3:**
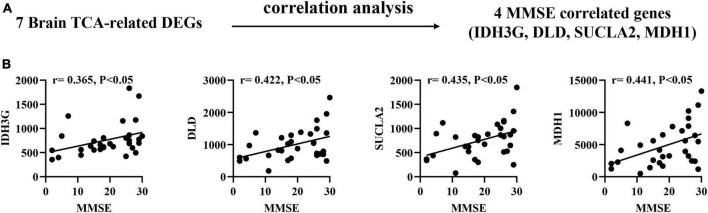
Correlation of brain TCA-related DEGs with mini-mental state examination (MMSE) in AD brain tissues. **(A)** Correlation analysis gene determination process **(B)** Pearson correlation coefficients (*r*) and corresponding *P*-values < 0.05 were displayed at the top of each plot. *X*-axis shows MMSE score, *y*-axis indicates relative expression abundance of each gene.

To confirm the association between brain biomarkers and cognitive decline, we performed correlation analysis between mRNA expression of related genes and MMSE scores in AD patients (GSE1297: Ctrl = 9, MCI = 22; Figure 3A). Pearson correlation analysis found that the IDH3G, DLD, SUCLA2, MDH1 showed moderate positive correlations with MMSE scores (*r* = 0.365–0.441, *p* < 0.05; Figure 3B), suggesting that their expression can effectively track the progression of cognitive decline.

### The mRNA expression levels of TCA cycle genes in peripheral blood of patients with MCI and AD and Ctrl

**FIGURE 4:**
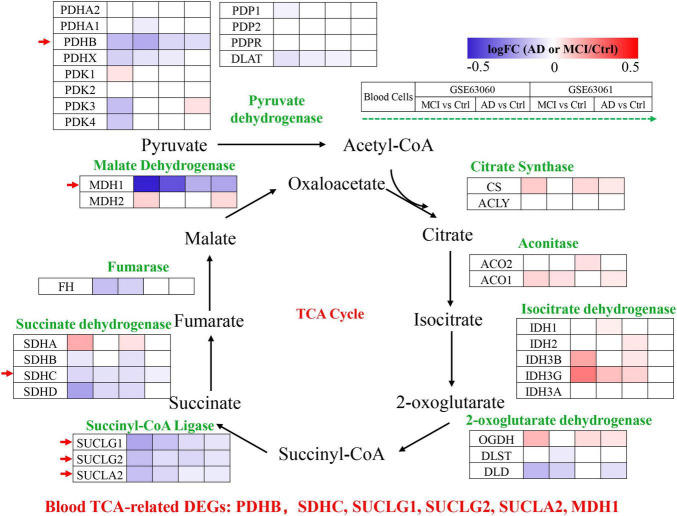
Tricarboxylic acid (TCA) cycle related genes were significantly differentially expressed in AD peripheral blood cells. The increased (red) or decreased (blue) level of proteins in MCI vs. Ctrl or AD vs. Ctrl (as indicated by the green arrow, GSE63060, GSE63061 from left to right, *P* < 0.05). Red arrows indicate genes that were consistently differentially expressed in GSE63060 and GSE63061.

Peripheral blood cells transcriptomes were obtained from GSE63060 (Ctrl = 104, MCI = 80, AD = 145) and GSE63061 (Ctrl = 134, MCI = 109, AD = 139), which were two large European blood cells RNA expression datasets. The expression of TCA cycle genes in the peripheral blood of AD patients were shown in Figure 4 and Table 2, with 16 genes significantly reduced and 11 genes significantly increased, which may be affected by the complex microenvironment of the peripheral system. Specifically, citrate synthase (CS), aconitase (ACO1, ACO2) and isocitrate dehydrogenase (IDH1, IDH2, IDH3A, IDH3B) were significantly increased. Pyruvate dehydrogenase (PDHB), succinyl-CoA ligase (SUCLG1, SUCLG2, SUCLA2), succinate dehydrogenase (SDHC), and malate Dehydrogenase (MDH1) showed consistent decrease in peripheral blood cells of MCI and AD in both GSE63060 and GSE63061 cohorts, which may be good peripheral blood biomarkers for early diagnosis of AD.

**TABLE 2:** The mRNA expression levels of TCA cycle genes in peripheral blood cells of patients with MCI and AD and Ctrl.

Blood cells	GSE63060						GSE63061					
	MCI vs. Ctrl			AD vs. Ctrl			MCI vs. Ctrl			AD vs. Ctrl		
Gene	logFC	*p*-value	adj *p*-value	logFC	*p*-value	adj *p*-value	logFC	*p*-value	adj *p*-value	logFC	*p*-value	adj *p*-value
**Pyruvate dehydrogenase**												
PDHA2	0.010	2.59E-01	5.16E-01	-0.008	3.15E-01	6.73E-01	-0.008	1.14E-01	3.74E-01	0.002	6.63E-01	8.86E-01
PDHA1	-0.037	8.72E-02	2.57E-01	-0.042	1.57E-02	1.16E-01	0.004	8.50E-01	9.50E-01	0.006	7.26E-01	9.13E-01
PDHB	-0.149	4.64E-05	5.60E-04	-0.187	1.48E-08	1.08E-06	-0.091	2.60E-04	9.11E-03	-0.077	2.33E-03	3.15E-02
PDHX	-0.103	1.13E-06	2.34E-05	-0.063	9.57E-04	1.31E-02	-0.045	3.59E-03	4.40E-02	-0.025	9.70E-02	3.73E-01
PDK1	0.057	4.39E-03	2.59E-02	0.030	7.99E-02	3.38E-01	0.001	9.47E-01	9.83E-01	0.007	5.20E-01	8.20E-01
PDK2	0.000	9.88E-01	9.96E-01	-0.007	4.91E-01	8.03E-01	0.013	3.78E-01	6.86E-01	0.002	8.83E-01	9.68E-01
PDK3	-0.146	7.89E-05	8.83E-04	0.050	5.40E-02	2.67E-01	0.041	5.90E-02	2.54E-01	0.060	1.50E-02	1.17E-01
PDK4	-0.122	1.12E-03	8.41E-03	-0.055	1.11E-01	4.07E-01	-0.060	9.48E-02	3.37E-01	-0.017	6.23E-01	8.69E-01
PDP1	-0.031	1.90E-03	1.30E-02	-0.013	1.66E-01	5.00E-01	-0.005	7.05E-01	8.87E-01	-0.010	4.34E-01	7.66E-01
PDP2	-0.011	3.29E-01	5.91E-01	-0.008	4.28E-01	7.63E-01	-0.011	1.72E-01	4.64E-01	-0.009	8.91E-02	3.55E-01
PDPR	0.021	1.09E-01	2.99E-01	0.010	3.85E-01	7.32E-01	-0.005	5.20E-01	7.91E-01	-0.005	5.46E-01	8.32E-01
DLAT	-0.067	5.84E-04	4.85E-03	-0.040	1.85E-02	1.30E-01	-0.030	3.26E-02	1.78E-01	-0.024	7.96E-02	3.33E-01
**Citrate synthase**												
CS	0.099	3.48E-04	3.11E-03	0.016	5.35E-01	8.29E-01	0.075	5.45E-03	5.76E-02	0.046	4.74E-02	2.44E-01
ACLY	0.057	5.99E-02	1.97E-01	0.039	1.64E-01	4.98E-01	0.028	1.44E-01	4.23E-01	0.021	2.68E-01	6.26E-01
**Aconitase**												
ACO1	0.084	2.39E-04	2.25E-03	0.061	1.98E-03	2.34E-02	0.026	1.31E-01	4.01E-01	0.044	7.21E-03	7.00E-02
ACO2	0.037	2.28E-01	4.78E-01	0.005	8.57E-01	9.60E-01	0.061	4.01E-03	4.71E-02	0.015	4.78E-01	7.94E-01
**Isocitrate dehydrogenase**												
IDH1	-0.048	1.35E-01	3.43E-01	0.037	4.75E-02	2.46E-01	-0.011	4.91E-01	7.71E-01	0.025	2.70E-01	6.28E-01
IDH2	0.03	3.56E-01	6.17E-01	-0.015	6.07E-01	8.62E-01	0.048	1.46E-02	1.07E-01	0.03	9.51E-02	3.68E-01
IDH3A	0.008	7.38E-01	8.82E-01	-0.02	3.32E-01	6.89E-01	0.0086	5.68E-01	8.19E-01	-0.016	2.54E-01	6.11E-01
IDH3B	0.174	9.53E-09	4.26E-07	0.049	8.94E-02	3.62E-01	0.05	2.88E-02	1.66E-01	-0.006	7.83E-01	9.34E-01
IDH3G	0.262	3.93E-10	3.13E-08	0.129	3.97E-04	6.47E-03	0.089	7.68E-04	1.74E-02	0.047	5.83E-02	2.78E-01
**2-oxoglutarate dehydrogenase**												
OGDHL	N/A	N/A	N/A	N/A	N/A	N/A	N/A	N/A	N/A	N/A	N/A	N/A
OGDH	0.141	1.69E-04	1.68E-03	0.047	1.40E-01	4.62E-01	0.069	3.66E-03	4.44E-02	0.055	8.00E-03	7.49E-02
DLST	-0.017	4.34E-01	6.84E-01	-0.046	1.10E-02	8.95E-02	-0.008	6.24E-01	8.49E-01	-0.0045	7.60E-01	9.25E-01
DLD	-0.158	2.27E-08	8.73E-07	-0.108	6.49E-06	2.07E-04	-0.039	8.51E-02	3.16E-01	-0.07	8.04E-04	1.48E-02
**Succinyl-CoA ligase**												
SUCLA2	-0.141	2.18E-07	5.75E-06	-0.089	2.28E-04	4.14E-03	-0.04	2.44E-02	1.49E-01	-0.045	1.77E-02	1.30E-01
SUCLG1	-0.197	6.50E-13	2.62E-10	-0.135	4.03E-10	4.66E-08	-0.077	1.40E-04	6.53E-03	-0.059	3.41E-03	4.11E-02
SUCLG2	-0.142	1.32E-05	1.89E-04	-0.076	1.55E-02	1.15E-01	-0.09	1.04E-04	5.52E-03	-0.058	9.54E-03	8.49E-02
**Succinate dehydrogenase**												
SDHA	0.163	1.93E-07	5.20E-06	0.057	5.54E-02	2.71E-01	0.058	6.30E-03	6.32E-02	0.025	1.96E-01	5.39E-01
SDHB	-0.053	3.65E-02	1.37E-01	0.008	7.33E-01	9.16E-01	-0.067	2.36E-04	8.59E-03	-0.009	6.07E-01	8.63E-01
SDHC	-0.089	6.69E-05	7.66E-04	-0.065	4.74E-04	7.49E-03	-0.068	2.18E-06	6.36E-04	-0.036	9.77E-03	8.63E-02
SDHD	-0.209	2.03E-05	2.75E-04	-0.087	4.42E-02	2.36E-01	-0.084	3.39E-03	4.23E-02	-0.012	6.54E-01	8.82E-01
**Fumarase**												
FH	-0.143	6.50E-04	5.30E-03	-0.105	5.33E-03	5.16E-02	0.011	5.39E-01	8.03E-01	0.022	1.94E-01	5.36E-01
**Malate dehydrogenase**												
MDH1	-0.499	3.29E-13	1.71E-10	-0.389	4.35E-11	6.46E-09	-0.18	1.15E-05	1.71E-03	-0.201	1.51E-06	1.43E-04
MDH2	0.089	1.12E-02	5.51E-02	0.06	6.28E-02	2.93E-01	0.035	1.71E-01	4.63E-01	0.074	1.87E-03	2.69E-02

Peripheral blood cells transcriptomes were obtained from GSE63060 (Ctrl = 104, MCI = 80, AD = 145) and GSE63061 (Ctrl = 134, MCI = 109, AD = 139). Ctrl, normal cognition controls group; MCI, mild cognitive impairment; Log2FC, log2 fold change.

**FIGURE 5:**
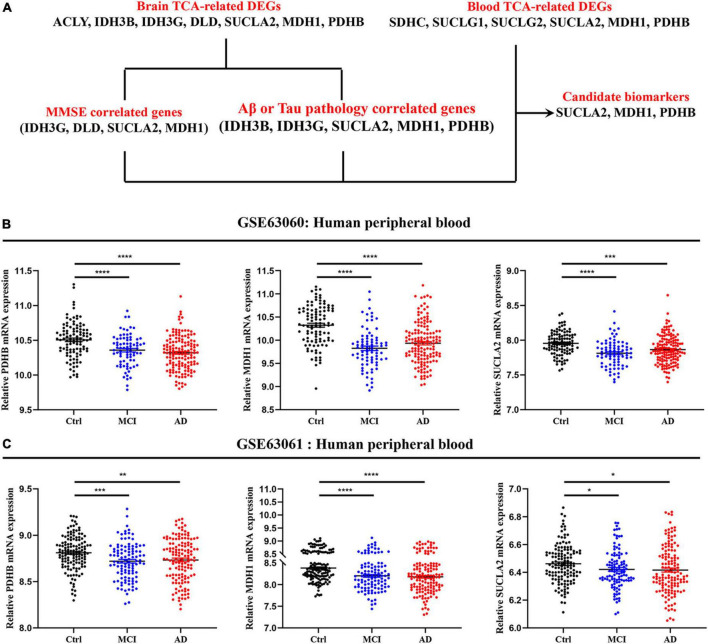
Expression level of candidate biomarkers in peripheral blood of AD patients. **(A)** Screening and validation process for candidate biomarkers. **(B,C)** Expression level of candidate biomarkers in peripheral blood of MCI and AD patients. Data were shown as mean ± SEM. *P < 0.05, **P < 0.01, ***P < 0.001, and ****P < 0.0001.

The mRNA expression of TCA cycle genes in AD patients was systematically revealed by brain and peripheral blood cells transcriptome data, and combined with MMSE, Aβ/Tau correlation analysis to confirm that SUCLA2, MDH1, and PDHB genes may be candidate biomarkers that can effectively track the pathological and clinical manifestations of AD patients (Figure 5A). By analyzing the transcriptome data of GSE63060 and GSE63061 peripheral blood cells, we found that pyruvate dehydrogenase (PDHB), succinyl-CoA ligase (SUCLA2) and malate dehydrogenase (MDH1) showed consistent decrease in peripheral blood cells of MCI and AD (Figures 5B, C), which were consistent with the trend in the central system.

## Discussion

Energy metabolism disorder is one of the earliest symptoms in AD and MCI, and it may precede the appearance of clinical cognitive impairment (Cunnane et al., 2011; Ding et al., 2013). Positron emission tomography (PET) imaging combined with 18F-2-deoxy-2-fluoro-d-glucose have shown that regional glucose utilization in AD patients is significantly decreased, and it was significantly correlated with MMSE scores, indicating that cognitive impairment was closely related to metabolic impairment in AD (de Leon et al., 1983). The susceptible areas of glucose metabolism decline in the brain of AD patients, including the posterior cingulate, parietal, and temporal cortices (Reiman et al., 1996; Small et al., 2000). Here, we systematically analyzed the expression of TCA cycle-related genes through large-scale mRNA expression profiling data in AD brain tissues, and showed that the overall expression of TCA cycle-related genes was decreased, suggesting that the TCA cycle was impaired in AD brain. Then, correlation analysis of Aβ, Tau and MMSE was performed to identify the molecules closely related to AD pathology and clinical manifestations. Finally, combined with the transcriptomics of peripheral blood cells from multiple AD cohorts, we found that PDHB, SUCLA2, and MDH1 can be used as candidate biomarkers for early diagnosis of AD.

Previous studies have shown that TCA cycle related genes were significantly decreased in AD brain (Bubber et al., 2005; Sang et al., 2022). Glucose is the main energy source for the brain, PDHC can convert pyruvate oxidative decarboxylation to acetyl-CoA, and reduced levels of PDHC result in less carbon entering the TCA cycle (Garabadu et al., 2019). Decreased PDHC leads to decreased acetyl-CoA production, which is a necessary raw material for acetylcholine production, and the correlation between acetyl-CoA and acetylcholine has been well established (Perry et al., 1980), so that decreased PDHC leads to decreased acetylcholine production. The loss of acetylcholine, which leads to cholinergic neuron loss, may further contribute to the loss of TCA cycle enzymes. TCA cycle enzymes deficiency leads to an increase in reactive oxygen species (ROS), which accelerates neurodegeneration (Klivenyi et al., 2004). In addition, pyruvate dehydrogenase deficiency is known to cause abnormal mitochondrial metabolism, lactate accumulation, and thus neuropathy (Patel et al., 2012). Here, we found that pyruvate dehydrogenase related genes PDHA1, PDHB, PDHX, DLAT showed consistent down-regulation in AD brain and blood cells, suggesting that pyruvate dehydrogenase deficiency may participate in AD-like nerve injury by regulating acetyl-CoA production leading to TCA cycle and acetylcholine production disorder.

Alpha-ketoglutarate dehydrogenase complex (αKGDHc), a key rate-limiting enzyme in the TCA cycle, can convert alpha-ketoglutarate, coenzyme A and NAD + to succinyl-coenzyme A, NADH and CO2, and its activity is significantly reduced in AD (Sheu et al., 1994; Stempler et al., 2014). Cholinergic neurons are crucial for αKGDHc defects are extremely sensitive, and αKGDHc mutations lead to increased ROS production and plays an important role in neurotoxicity (Sheu and Blass, 1999). Studies have shown that the decreased activity of αKGDHc is significantly correlated with cognitive decline in AD (Bubber et al., 2005; Shi et al., 2008). DLD deficient mice have aggravated mitochondrial damage and reduced neurogenesis (Klivenyi et al., 2004), and DLD inhibition can exacerbate Tau phosphorylation, thereby promoting the development of AD pathology (Ahmad, 2018). Dihydrolipoyllysine-residue succinyltransferase component of 2-oxoglutarate dehydrogenase complex (DLST) deficiency can exacerbate amyloid protein deposition and memory damage in amyloid-beta precursor protein (APP)/PS1 mice (Dumont et al., 2009). In addition, the expression of OGDHL was significantly reduced in 3xTg-AD mice, while overexpression of OGDHL could reduce neuroinflammation, amyloid plaques, and Tau phosphorylation (Yao et al., 2022). Here, we found that DLD was significantly decreased in the brain and blood cells of AD patients, and significantly positively correlated with MMSE scores, suggesting that it may not only be involved in the pathological development of AD, but also may mediate clinical cognitive impairment.

Succinyl-CoA ligase (SCL) catalyzes substrate level phosphorylation, and the related subunits are significantly reduced in AD, especially SUCLA2 (Sang et al., 2022). Succinyl-CoA and Glycine, as precursors, are involved in the synthesis of heme (Ponka, 1999). The levels of enzymes involved in the TCA cycle decrease, leading to a decrease in succinyl-CoA, which in turn decreases the ability to synthesize heme (Atamna and Frey, 2007). When heme deficiency occurs, key cellular pathological phenotypes of AD are produced (Atamna et al., 2001, 2002; Scheuermann et al., 2001), such as decreased levels of complex IV, iron deposition, mitochondrial dysfunction, APP dimerization, and neuronal death. Here, we found that SUCLA2 was significantly decreased in AD brain and blood cells, and correlated with Aβ, Tau and MMSE scores, suggesting that SUCLA2 may be closely related to the pathological development and cognitive impairment of AD, and it may be a potential diagnostic and therapeutic target.

Malate dehydrogenase is involved in two major metabolic processes, namely malate-aspartate shuttle and tricarboxylic acid cycle, which are essential for mitochondrial respiration and adenosine 5’-triphosphate (ATP) production (Friedrich et al., 1988). Previous studies have found that MDH1, as a hub gene, participates in the network regulation of AD and may be crucial for its pathological development (Wang et al., 2017; Liu et al., 2020). MDH1 deficiency can cause severe metabolic disorders, thereby promoting delayed neural development (Broeks et al., 2019). Here, we found that MDH1 was significantly decreased in AD, and correlated with the typical pathologies of AD, and parallel with clinical cognitive decline.

In sum, we systematically analyzed the expression of TCA cycle related genes in the central and peripheral of AD, revealing molecules closely related to AD pathological development and clinical cognitive decline, providing valuable resources for further exploring the specific mechanisms between TCA cycle and AD progression. PDHB, SUCLA2, and MDH1 may be potential diagnostic and therapeutic targets.

## Data availability statement

The original contributions presented in this study are included in the article/supplementary material, further inquiries can be directed to the corresponding authors.

## Author contributions

HY and DJ: experimental design and manuscript-writing. HY, DJ, and FW: experimental methods and data analysis. All authors contributed to the article and approved the submitted version.

## Footnotes

1
http://www.alzdata.org/

2
www.mouseac.org

3
https://www.bioconductor.org/packages/release/bioc/html/limma.html

## References

[B1] Ahmad W. (2018). Dihydrolipoamide dehydrogenase suppression induces human tau phosphorylation by increasing whole body glucose levels in a *C. elegans* model of Alzheimer’s Disease. *Exp. Brain Res.* 236 2857–2866. 10.1007/s00221-018-5341-0 30056470

[B2] Atamna H. Frey W. II (2007). Mechanisms of mitochondrial dysfunction and energy deficiency in Alzheimer’s disease. *Mitochondrion* 7 297–310. 10.1016/j.mito.2007.06.001 17625988

[B3] Atamna H. Killilea D. Killilea A. Ames B. (2002). Heme deficiency may be a factor in the mitochondrial and neuronal decay of aging. *Proc. Natl. Acad. Sci. U.S.A.* 99 14807–14812. 10.1073/pnas.192585799 12417755 PMC137500

[B4] Atamna H. Liu J. Ames B. (2001). Heme deficiency selectively interrupts assembly of mitochondrial complex IV in human fibroblasts: revelance to aging. *J. Biol. Chem.* 276 48410–48416. 10.1074/jbc.M108362200 11598132

[B5] Barrett T. Wilhite S. Ledoux P. Evangelista C. Kim I. Tomashevsky M. (2013). NCBI GEO: archive for functional genomics data sets–update. *Nucleic Acids Res.* 41 D991–D995. 10.1093/nar/gks1193 23193258 PMC3531084

[B6] Broeks M. Shamseldin H. Alhashem A. Hashem M. Abdulwahab F. Alshedi T. (2019). MDH1 deficiency is a metabolic disorder of the malate-aspartate shuttle associated with early onset severe encephalopathy. *Hum Genet.* 138 1247–1257. 10.1007/s00439-019-02063-z 31538237

[B7] Bubber P. Haroutunian V. Fisch G. Blass J. Gibson G. (2005). Mitochondrial abnormalities in Alzheimer brain: mechanistic implications. *Ann. Neurol.* 57 695–703. 10.1002/ana.20474 15852400

[B8] Butterfield D. Halliwell B. (2019). Oxidative stress, dysfunctional glucose metabolism and Alzheimer disease. *Nat. Rev. Neurosci.* 20 148–160. 10.1038/s41583-019-0132-6 30737462 PMC9382875

[B9] Cunnane S. Nugent S. Roy M. Courchesne-Loyer A. Croteau E. Tremblay S. (2011). Brain fuel metabolism, aging, and Alzheimer’s disease. *Nutrition* 27 3–20. 10.1016/j.nut.2010.07.021 21035308 PMC3478067

[B10] de Leon M. Ferris S. George A. Christman D. Fowler J. Gentes C. (1983). Positron emission tomographic studies of aging and Alzheimer disease. *AJNR Am. J. Neuroradiol.* 4 568–571. 6410799 PMC8334899

[B11] Ding F. Yao J. Rettberg J. Chen S. Brinton R. (2013). Early decline in glucose transport and metabolism precedes shift to ketogenic system in female aging and Alzheimer’s mouse brain: implication for bioenergetic intervention. *PLoS One* 8:e79977. 10.1371/journal.pone.0079977 24244584 PMC3823655

[B12] Dumont M. Ho D. Calingasan N. Xu H. Gibson G. Beal M. (2009). Mitochondrial dihydrolipoyl succinyltransferase deficiency accelerates amyloid pathology and memory deficit in a transgenic mouse model of amyloid deposition. *Free Radic. Biol. Med.* 47 1019–1027. 10.1016/j.freeradbiomed.2009.07.008 19596066 PMC2761144

[B13] Friedrich C. Ferrell R. Siciliano M. Kitto G. (1988). Biochemical and genetic identity of alpha-keto acid reductase and cytoplasmic malate dehydrogenase from human erythrocytes. *Ann. Hum. Genet.* 52 25–37. 10.1111/j.1469-1809.1988.tb01075.x 3052244

[B14] Garabadu D. Agrawal N. Sharma A. Sharma S. (2019). Mitochondrial metabolism: a common link between neuroinflammation and neurodegeneration. *Behav. Pharmacol.* 30 642–652. 10.1097/FBP.0000000000000505 31625975

[B15] GBD 2016 Neurology Collaborators (2019). Global, regional, and national burden of neurological disorders, 1990-2016: a systematic analysis for the Global Burden of Disease Study 2016. *Lancet Neurol.* 18 459–480. 10.1016/S1474-4422(18)30499-X 30879893 PMC6459001

[B16] Hodson R. (2018). Alzheimer’s disease. *Nature* 559:S1. 10.1038/d41586-018-05717-6 30046078

[B17] Klivenyi P. Starkov A. Calingasan N. Gardian G. Browne S. Yang L. (2004). Mice deficient in dihydrolipoamide dehydrogenase show increased vulnerability to MPTP, malonate and 3-nitropropionic acid neurotoxicity. *J. Neurochem.* 88 1352–1360. 10.1046/j.1471-4159.2003.02263.x 15009635

[B18] Liu L. Wu Q. Zhong W. Chen Y. Zhang W. Ren H. (2020). Microarray analysis of differential gene expression in Alzheimer’s disease identifies potential biomarkers with diagnostic value. *Med. Sci. Monit.* 26:e919249. 10.12659/MSM.919249 31984950 PMC7001516

[B19] Marin-Garcia J. Ananthakrishnan R. Goldenthal M. (1998). Human mitochondrial function during cardiac growth and development. *Mol. Cell Biochem.* 179 21–26. 10.1023/a:1006839831141 9543345

[B20] Matarin M. Salih D. Yasvoina M. Cummings D. Guelfi S. Liu W. (2015). A genome-wide gene-expression analysis and database in transgenic mice during development of amyloid or tau pathology. *Cell Rep.* 10 633–644. 10.1016/j.celrep.2014.12.041 25620700

[B21] Patel K. O’Brien T. Subramony S. Shuster J. Stacpoole P. (2012). The spectrum of pyruvate dehydrogenase complex deficiency: clinical, biochemical and genetic features in 371 patients. *Mol. Genet. Metab.* 105 34–43. 10.1016/j.ymgme.2011.09.032 22079328 PMC3754811

[B22] Perry E. Perry R. Tomlinson B. Blessed G. Gibson P. (1980). Coenzyme A-acetylating enzymes in Alzheimer’s disease: possible cholinergic ‘compartment’ of pyruvate dehydrogenase. *Neurosci. Lett.* 18 105–110. 10.1016/0304-3940(80)90220-7 6133246

[B23] Ponka P. (1999). Cell biology of heme. *Am. J. Med. Sci.* 318 241–256. 10.1097/00000441-199910000-00004 10522552

[B24] Potapova I. El-Maghrabi M. Doronin S. Benjamin W. (2000). Phosphorylation of recombinant human ATP:citrate lyase by cAMP-dependent protein kinase abolishes homotropic allosteric regulation of the enzyme by citrate and increases the enzyme activity. Allosteric activation of ATP:citrate lyase by phosphorylated sugars. *Biochemistry* 39 1169–1179. 10.1021/bi992159y 10653665

[B25] Reiman E. Caselli R. Yun L. Chen K. Bandy D. Minoshima S. (1996). Preclinical evidence of Alzheimer’s disease in persons homozygous for the epsilon 4 allele for apolipoprotein E. *N. Engl. J. Med.* 334 752–758. 10.1056/NEJM199603213341202 8592548

[B26] Sang C. Philbert S. Hartland D. Unwin R. Dowsey A. Xu J. (2022). Coenzyme A-dependent tricarboxylic acid cycle enzymes are decreased in Alzheimer’s disease consistent with cerebral pantothenate deficiency. *Front. Aging Neurosci.* 14:893159. 10.3389/fnagi.2022.893159 35754968 PMC9232186

[B27] Scheltens P. De Strooper B. Kivipelto M. Holstege H. Chételat G. Teunissen C. (2021). Alzheimer’s disease. *Lancet* 397 1577–1590. 10.1016/S0140-6736(20)32205-4 33667416 PMC8354300

[B28] Scheuermann S. Hambsch B. Hesse L. Stumm J. Schmidt C. Beher D. (2001). Homodimerization of amyloid precursor protein and its implication in the amyloidogenic pathway of Alzheimer’s disease. *J. Biol. Chem.* 276 33923–33929. 10.1074/jbc.M105410200 11438549

[B29] Seifert F. Ciszak E. Korotchkina L. Golbik R. Spinka M. Dominiak P. (2007). Phosphorylation of serine 264 impedes active site accessibility in the E1 component of the human pyruvate dehydrogenase multienzyme complex. *Biochemistry* 46 6277–6287. 10.1021/bi700083z 17474719

[B30] Sheu K. Blass J. (1999). The alpha-ketoglutarate dehydrogenase complex. *Ann. N. Y. Acad. Sci.* 893 61–78. 10.1111/j.1749-6632.1999.tb07818.x 10672230

[B31] Sheu K. Cooper A. Koike K. Koike M. Lindsay J. Blass J. (1994). Abnormality of the alpha-ketoglutarate dehydrogenase complex in fibroblasts from familial Alzheimer’s disease. *Ann Neurol.* 35 312–318. 10.1002/ana.410350311 8122883

[B32] Shi Q. Xu H. Kleinman W. Gibson G. (2008). Novel functions of the alpha-ketoglutarate dehydrogenase complex may mediate diverse oxidant-induced changes in mitochondrial enzymes associated with Alzheimer’s disease. *Biochim. Biophys. Acta* 1782 229–238. 10.1016/j.bbadis.2007.12.008 18206986 PMC3106300

[B33] Shoshan-Barmatz V. Nahon-Crystal E. Shteinfer-Kuzmine A. Gupta R. (2018). VDAC1, mitochondrial dysfunction, and Alzheimer’s disease. *Pharmacol. Res.* 131 87–101. 10.1016/j.phrs.2018.03.010 29551631

[B34] Small G. Ercoli L. Silverman D. Huang S. Komo S. Bookheimer S. (2000). Cerebral metabolic and cognitive decline in persons at genetic risk for Alzheimer’s disease. *Proc. Natl. Acad. Sci. U.S.A.* 97 6037–6042. 10.1073/pnas.090106797 10811879 PMC18554

[B35] Sood S. Gallagher I. Lunnon K. Rullman E. Keohane A. Crossland H. (2015). A novel multi-tissue RNA diagnostic of healthy ageing relates to cognitive health status. *Genome Biol.* 16:185. 10.1186/s13059-015-0750-x 26343147 PMC4561473

[B36] Stempler S. Yizhak K. Ruppin E. (2014). Integrating transcriptomics with metabolic modeling predicts biomarkers and drug targets for Alzheimer’s disease. *PLoS One* 9:e105383. 10.1371/journal.pone.0105383 25127241 PMC4134302

[B37] Wang Z. Yan X. Zhao C. (2017). Dynamical differential networks and modules inferring disrupted genes associated with the progression of Alzheimer’s disease. *Exp. Ther. Med.* 14 2969–2975. 10.3892/etm.2017.4905 28966679 PMC5613183

[B38] Xu M. Zhang D. Luo R. Wu Y. Zhou H. Kong L. (2018). A systematic integrated analysis of brain expression profiles reveals YAP1 and other prioritized hub genes as important upstream regulators in Alzheimer’s disease. *Alzheimers Dement.* 14 215–229. 10.1016/j.jalz.2017.08.012 28923553

[B39] Yao L. Xu X. Xu Y. Li C. Xie F. Guo M. (2022). OGDHL ameliorates cognitive impairment and Alzheimer’s disease-like pathology via activating Wnt/β-catenin signaling in Alzheimer’s disease mice. *Behav. Brain Res.* 418:113673. 10.1016/j.bbr.2021.113673 34798170

